# Electron transport properties of graphene quantum dots with non-centro-symmetric Gaussian deformation

**DOI:** 10.1038/s41598-022-14251-5

**Published:** 2022-06-14

**Authors:** A. Poszwa

**Affiliations:** Faculty of Mathematics and Computer Science, University of Warmia and Mazury in Olsztyn, ul. Słoneczna 54, 10-710 Olsztyn, Poland

**Keywords:** Nanoscience and technology, Nanoscale devices, Electronic devices

## Abstract

A theoretical investigation on electron transport properties of rectangular graphene quantum dots (GQDs) with non-centro-symmetric out-of-plane Gaussian deformation of elliptic type is presented. Different levels of deformation are explored to estimate system geometry optimal for potential electronic applications. Electronic properties of deformed GQDs are studied in terms of local density of states (LDOS), band-gap opening and equilibrium ballistic conductance. In particular, it was observed that the symmetry of spatial LDOS structure is directly linked with the symmetry of properly defined local strain field (LSF) map, for a wide energy range. The relationship confirms qualitatively predictions obtained on the basis of the concept of a pseudomagnetic field, used in continuum models of graphene, including strain induced effects. The conductance spectra of deformed GQD as a device connected to semi-infinite graphene armchair nanoribbons as reservoirs are studied in a frame of tight-binding (TB) model in combination with non-equilibrium Green’s-functions technique (NEGF).

## Introduction

Geometry-induced effects in 2D materials has emerged in recent years as an active area of research due to potential use for electronic purposes. The coupling of geometrical and electronic properties seems to be a promising tool for the design of electronic devices with a desired electric transport properties. A representative example of the interplaying between geometry and the electronic band structure in graphene systems are graphene nanoribbons (GNRs) and carbon nanotubes (CNTs)^1,2^. In the former, the electronic states largely depend on the edge structure and on the width of the GNR, in the latter the electrical properties depend on the shape of the edges and on the diameter of the CNT. Dependence of the electron transport on the geometry has been studied theoretically and experimentally in many structures characterized by non-uniform geometry, in particular in the ballistic regime. The motion of charge carriers can be considered ballistic, when the constriction length of the device is on the scale of mean-free path length of the material. One of the most known devices of this type is the geometric diode^3,4^. The geometric diode is a ballistic transport device providing electric rectification due to geometrical asymmetry. In many materials, energy bands exhibit a discrete number of inequivalent local minima or maxima for specific values of momenta, usually known as *valleys*^5,6^. These valleys seem to be ideal candidates for components of a binary variable or pseudospin. In the case of graphene in particular, different schemes have been proposed to achieve valley-electronic filtering depending on geometrical deformation, also referred to as valley polarization, i.e. generating a charge current composed of electron states from only one valley. Strain-induced effects in graphene has been the topic of a large number of theoretical works aimed at understanding the impact of controlled geometrical deformations on electronic properties^7–12^. Most of theoretical study focus on strain produced by a centrosymmetric Gaussian out-of-plane deformation^12–15^. They have been already produced experimentally on suitable substrates^16^ and also with STM methods^17^. Confining states within deformed GNR, symmetry properties of LDOS, conductance spectra and pseudospin polarization have been studied in the frame of TB approximation^18^. The effects of an external electric field, the influence of edge roughness on conduction properties of deformed GNRs with centrosymmetric Gaussian bump have been studied using TB model and NEGF transport formalism^19^.

**Figure 1:**
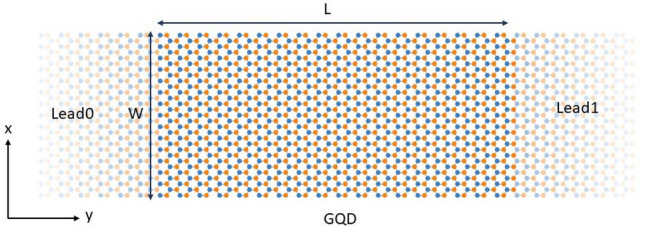
The schematic view of the armchair graphene nanoribbon system. The linear sizes of the rectangular GQD are defined as $W=(M-1)\sqrt{3}/2\times a_0$ and $L=(3N-2)\times a_0/2$, where $a_0=1.42$ Å is the carbon-carbon distance. The numbers *M*, *N* are integers.

**Figure 2:**
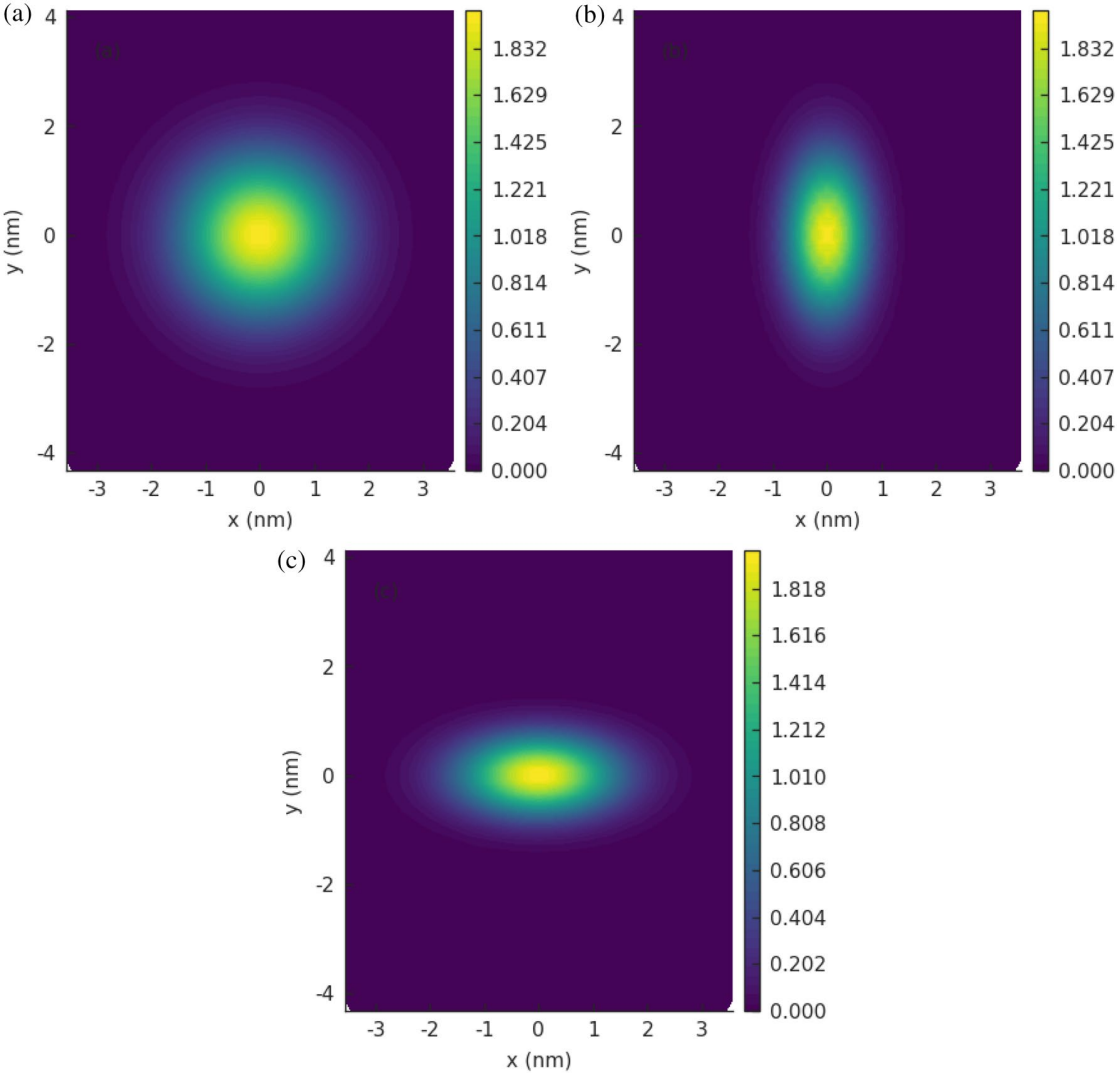
Rectangular GQDs (M=60, N=40) with elliptic Gaussian bumps in the center: (**a**) $h_G=2 \, \text {nm}, \sigma _x=1\, \text {nm}, \sigma _y=1\,\text {nm}$; (**b**) $h_G=2\,\text {nm}, \sigma _x=0.5\,\text {nm}, \sigma _y=1\,\text {nm}$; (**c**) $h_G=2\,\text {nm}, \sigma _x=1\, \text {nm}, \sigma _y=0.5\, \text {nm}$.

**Figure 3:**
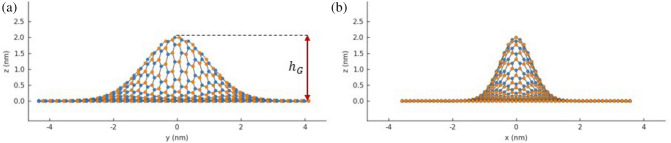
Profiles of elliptic Gaussian bump illustrated in Fig. 2b: (**a**) *zy*; (**b**) *zx*.

In this work, I investigate the impact of anisotropic Gaussian deformation on static electronic properties of GQDs and on electron transport in armchair graphene system given in Fig. 1. I consider three types of Gaussian bumps illustrated in Fig. 2. The example profiles of GQD with elliptic Gaussian deformation are given in Fig. 3. The main goal of this paper is to estimate optimal geometric configuration of the central part of the structure GNR-GQD-GNR giving appropriate profile of conductance as function of energy, suitable for electronic purposes. Considering this system as a main structural element of the nanotransistor one expect to obtain high conductance in *on* state and low conductance in *off* state. The necessary condition to achieve such a conductance property is the existence of sufficiently large energy gap and relatively high conductance outside the gap. We note that recent studies on electron transport properties of GNRs, focused on the energy gap opening mechanism^19^ and valley filtering mechanism^6^, are limited to centrosymmetric Gaussian bumps or pure Gaussian folds. In particular, it has been shown that centrosymmetric Gaussian deformation strongly modifies the electronic properties of all types of ribbon structures and leads to a strong reduction of electron transmission in high-energy regions^19^. Investigations presented in this paper show that for the systems with comparable number of transmission modes as considered in the paper^19^, fundamental requirements related to transport properties (large transport gap and good conductance for higher energies) can be satisfied simultaneously for properly deformed GQD as an effect of geometric anisotropy, modeled by Gaussian deformation, of an elliptic type.

## Model

In order to investigate transport properties of any system, it is convenient to use some *discrete* representation of the Hamiltonian, such as that obtained within the finite difference method or in TB approximation. The *discrete* representation allows for easily determination of contact operators describing couplings between the device and leads. Also, using such a representation, the calculation of appropriate Green’s functions reduces to performing numerical operations directly on matrices. In the case of graphene systems, a commonly used and the most suitable for transport purposes is a single-orbital TB model for the $\pi$-electron network. The single orbital is the $p_z$ atomic orbital of carbon, that is decoupled from the in-plane $\sigma$ orbitals (formed by *s*, $p_x$ and $p_y$ orbitals). Using the first-nearest-neighbors (1NN) approximation the single-orbital, *semi-empirical* TB Hamiltonian reads

1 $$\begin{aligned} \mathsf H=-\sum _{i,j\in 1NN}t_{ij}|i\rangle \langle j|+\sum _{i}\varepsilon _i|i\rangle \langle i|, \end{aligned}$$

where $\varepsilon _i$ is the on-site energy and $t_{ij}$ is the transfer (hopping) energy. For a bulk system, in order to simplify the formulation, on-site energies are conventionally set as the reference energy $\varepsilon _i=0$. Using the restriction of electron hopping only between first-nearest neighbors, the hopping parameters are supposed to be all equal and reduced to $t_{ij}=t_0=2.8$eV. However, the representation of a finite system by an atomistic model ultimately requires the introduction of a limited simulation domain (at least in one spatial direction), of which the surface needs to be treated with a specific boundary condition (BC)^20–26^. One of the choice in representing a finite simulation domain is the abrupt termination of the simulation domain with a hard-wall BC. Such abrupt termination in the atomistic basis set results in the creation of dangling bonds^25^. The dangling bonds will form surface states that typically cover a broad energy range and often litter the central energy region of the fundamental band gap. To model GNRs in a more realistic treatment we can the easiest assume that edge carbon atoms are passivated with hydrogen, so that there are no dangling bonds at the edge and parasitic boundary condition induced states are eliminated. On the other hand, this treatment requires some modification of TB parameters corresponding to edge atoms since the presence of C–H bonds introduces carbon atoms on edge with a different nature. One should be noted here that the edge effects can be taken into account simply by modifying the transfer energy $t_{ij}$ and the on-site energy $\varepsilon _i$ in the Hamiltonian (Eq. 1)^23^. However, as it has been shown in the paper^27^ the modification of band structure introduced by edge corrections is small, though some of metallic armchair nanoribbons become semiconducting. This, however, works to the advantage of our search, as we are looking for effects leading, among other, to increasing of the energy gap. For this reason, further calculations are performed using the Hamiltonian (Eq. 1) without any modifications, related to edge effects. To obtain band structure and Green’s functions of leads, periodic boundary conditions along the ribbon axes are supposed with properly defined unit cell. The energy spectrum and Green’s function of the device region are calculated with open boundary conditions imposed at zigzag edges of central GQD, resulting from contacts self-energies described by non-hermitian operators.

In the presence of the Gaussian deformation the system is described by the Hamiltonian (Eq. 1), in which the hopping energy between two lattice sites *i*th and *j*th is modified to^12,19,28^

2 $$\begin{aligned} t_{ij}=t_0e^{-\beta (d_{ij}/a_0-1)}, \end{aligned}$$

where $a_0=1.42$ Å is the carbon-carbon distance and the dimensionless coefficient $\beta =3.37$ is defined by the strain theory^28^. $t_0=2.8$eV is defined above hopping energy between the two nearest sites in the unstrained region and $d_{ij}=\sqrt{(x_i-x_j)^{2}+(y_i-y_j)^{2}+(z_i-z_j)^{2}}$ is the distance between the *i*th and *j*th sites. The on-site energies are supposed to be unchanged and $\varepsilon _i=0$, for all sites. At this point, one should be noted that hopping renormalization given in Eq. (2) is commonly used in theoretical investigation of strain-induced effects in graphene but it can not be treated as a general rule. For instance, in TB models with $sp^{3}d^{5}s^{*}$ parametrization also diagonal corrections are considered^29,30^. The source of these corrections however is the presence of *d*-orbitals in the TB model that is not the case of graphene.

The coordinate *z* of atoms within an elliptic Gaussian deformed region is defined as

3 $$\begin{aligned} z(x,y)=h_Ge^{-[(x-x_0)^{2}/2\sigma ^{2}_x+(y-y_0)^{2}/2\sigma ^{2}_y]}, \end{aligned}$$

where in general, the standard deviations $\sigma _x$ and $\sigma _y$ of the Gaussian shape are different for *x*- and *y*-directions. The Gaussian bump defined in Eq. (3) is centred on the position with coordinates $x_0, y_0$. In our study $x_0=0, y_0=0$, which coincides with the origin of the coordinate system chosen at the center of the GQD. The GQD is taken as a finite rectangular piece of armchair GNR limited by the zigzag edges (see Fig. 1). The geometrical size of the GQD is defined using commonly known convention^31^. For the system given in Fig. 1, the width *W* of the GQD is given by the integer *M*, where *M* stands for the number of dimer lines counted along the zigzag edge, $W=(M-1)\sqrt{3}/2\times a_0$ and the length *L* of the GQD is determined by the integer *N*, where *N* is the number of the zigzag lines along the armchair edge, $L=(3N-2)\times a_0/2$. In further part of the work the linear sizes of the GQDs are given in terms of the integers *M* and *N*.

The quantum transport properties are studied using TB model coupled to the NEGF formalism^32,33^. As we can see in Fig. 1, all system is divided into three parts: semi-infinite left lead, the central region (device) and semi-infinite right lead. The leads are taken in the form of armchair GNRs with the width equal to the width of the central region. The device has the form of rectangular GQD with elliptic Gaussian bump, in the center. The length of the device is taken large enough to ensure smooth connection to the leads, that are supposed to be strictly periodic structures, in the transport direction. The Hamiltonian matrix of the entire system, in TB representation, can be written in the block form

4 $$\begin{aligned} H=\left[ \begin{array} {ccc} H_L &{} T^{\dagger }_{DL} &{} 0\\ T_{DL} &{} H_D &{} T_{DR} \\ 0 &{} T^{\dagger }_{DR} &{} H_R\\ \end{array}\right] , \end{aligned}$$

where $H_{L(R)}$ and $H_D$ mean the Hamiltonian matrix of the left (right) lead and the device Hamiltonian, respectively. The coupling between the device and the left (right) lead is described by the operator $T_{DL(R)}$. According to the NEGF method the electron transmission function at energy *E* is given by^33^

5 $$\begin{aligned} T(E)=\text {Tr}(\Gamma _LG_D\Gamma _RG_D^{\dagger }), \end{aligned}$$

where $\Gamma _{L(R)}=i(\Sigma _{L(R)}-\Sigma ^{\dagger }_{L(R)})$ and $\Sigma _{L(R)}=T_{DL(R)}G^{(0)}_{L(R)}T^{\dagger }_{DL(R)}$ denotes self-energy matrix, representing the effect of coupling to the left (right) lead. $G^{(0)}_{L(R)}$ represents the Green’s function of the isolated left (right) lead. $G_D$ is the Green’s function of the device including the coupling to reservoirs

6 $$\begin{aligned} G_D=[E+i\eta -H_D-\Sigma _L-\Sigma _R]^{-1}, \end{aligned}$$

where $\eta$ is an infinitesimal positive number introduced to incorporate appropriate boundary conditions for retarded Green’s function^33^. The LDOS at the *j*th lattice site is given by^33^

7 $$\begin{aligned} D(E,\mathbf{r} _j)=-\frac{1}{\pi }\text {Im}[G_D(j,j,E)], \end{aligned}$$

where $G_D(j,j,E)$ is relevant matrix element of the retarded Green’s function given in Eq. (6). The Green’s functions of the semi-infinite leads and the device region were calculated using recursive techniques presented in^34^. We note that equilibrium conductance is obtained from transmission simply as $G(E)=(e^{2}/h)T(E)$. We will consider in this paper transport properties of armchair GNR systems classified into three groups, based on the classification scheme applied to perfect armchair GNRs: $M=3p+2, 3p+1, 3p$ where *p* is an integer number. The first group contains semimetalic GNRs, the two remaining groups correspond to semiconducting GNRs. In the present study *M* is taken as an odd number.

## Results and discussion

**Figure 4:**
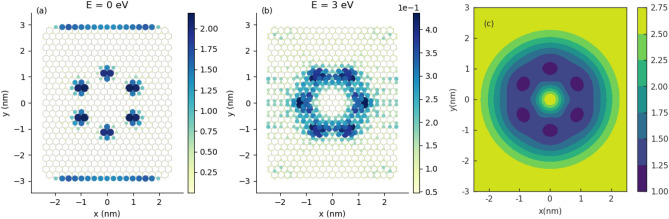
The LDOS maps for GQD with centrosymmetric Gaussian bump for electron energies: (**a**) $E=0$ and (**b**) $E=3$eV; (**c**) local strain field (LSF) corresponding to the geometry: $h_G=2 \, \text {nm}, \sigma =1\, \text {nm}$. The LDOS have been generated with the help of Pybinding Python package^35^.

In Fig. 4a,b are given LDOS maps for GQD with centrosymmetric Gaussian deformation ($h_G=2\,\text {nm}, \sigma _x=\sigma _y=1\,\text {nm}$) corresponding to the electron states with energies $E=0$ and $E=3$ eV, respectively. We note that one of the most peculiar properties of the graphene nanoribbons is that the states at $E=0$ are spatially located on the zigzag edges of the ribbon^20–22^. This property remains valid for a finite unstrained system, like the rectangular GQD, considered in this study. We can see in Fig. 4a that for deformed system, the low-energy states are localized partially at the zigzag edges but also in the center of the dot inside the bump with characteristic flower-like structure. The *petals* of this *flower* are located on armchair directions of the GQD. On the other hand, we can see in part b of Fig. 4 that the states with higher energy occupy the zigzag directions of the GQD and partially are localized outside the bump. This can be explained by noting that at low energy there is strong confinement inside the bump near the strain maximum. At higher energy, confinement is weaker as more states are found outside the bump. In the present study, the flower-like structure of LDOS with $60^{\circ }$ symmetry reflects the presence of local extrema in the local strain field. Details of calculations are given in the “Appendix”. We can see that low (high) energy states occupy mostly minima (maxima) in the LSF distribution. The map of LSF is given in Fig. 4c. The symmetry analysis is qualitatively consistent with results obtained using the concept of strain-induced pseudomagnetic field, introduced in continuum model of graphene^6^.

**Figure 5:**
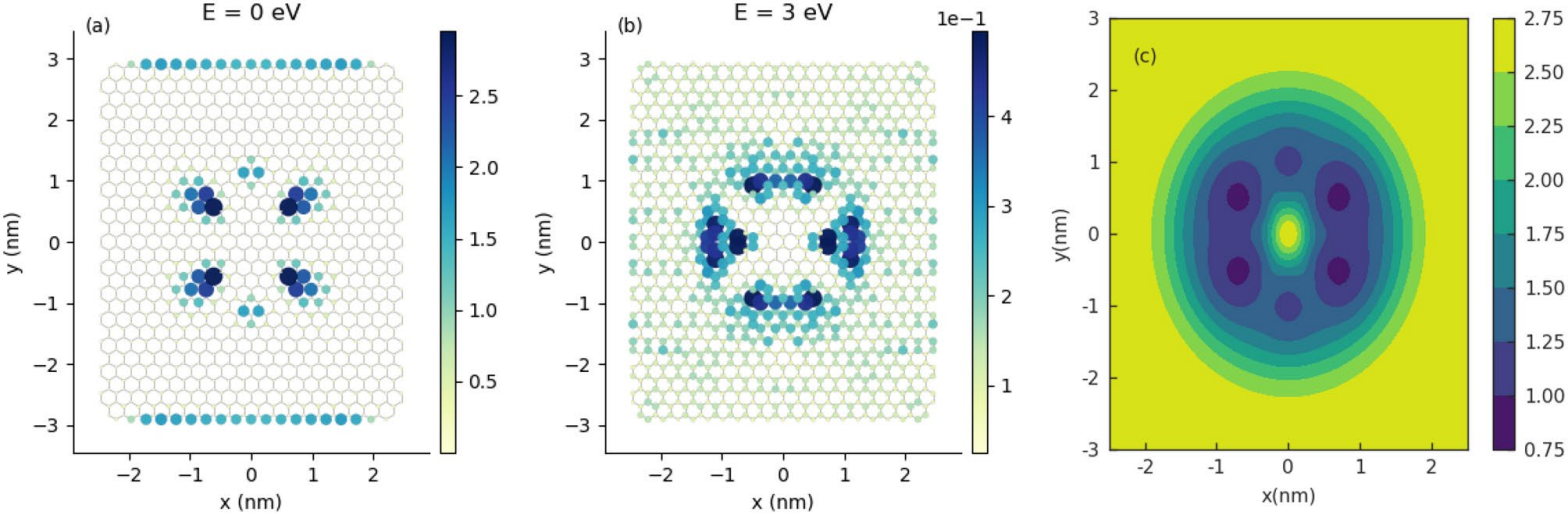
The LDOS maps for GQD with non-centrosymmetric Gaussian bump for electron energies: (**a**) $E=0$ and (**b**) $E=3$ eV; (**c**) local strain field (LSF) corresponding to the geometry: $h_G=2\text {nm}, \sigma =1\,\text {nm}, \sigma _x=0.8\,\text {nm}$.

In Fig. 5a,b are given LDOS maps for GQD with non-centrosymmetric Gaussian deformation ($h_G=2\,\text {nm}, \sigma _x=0.8\,\text {nm}, \sigma _y=1\text {nm}$) corresponding to the electron states with energies $E=0$ and $E=3$ eV, respectively. In particular, we can see in Fig. 5a that the zigzag edge zero-energy states decreases gradually with increasing of anisotropy of deformed region with approximately fixed strain. We can also see in Fig. 5c that directions of LSF extrema are rotated relatively armchair and zigzag directions, that is a consequence of anisotropy introduced by the deformation geometry.

**Figure 6:**
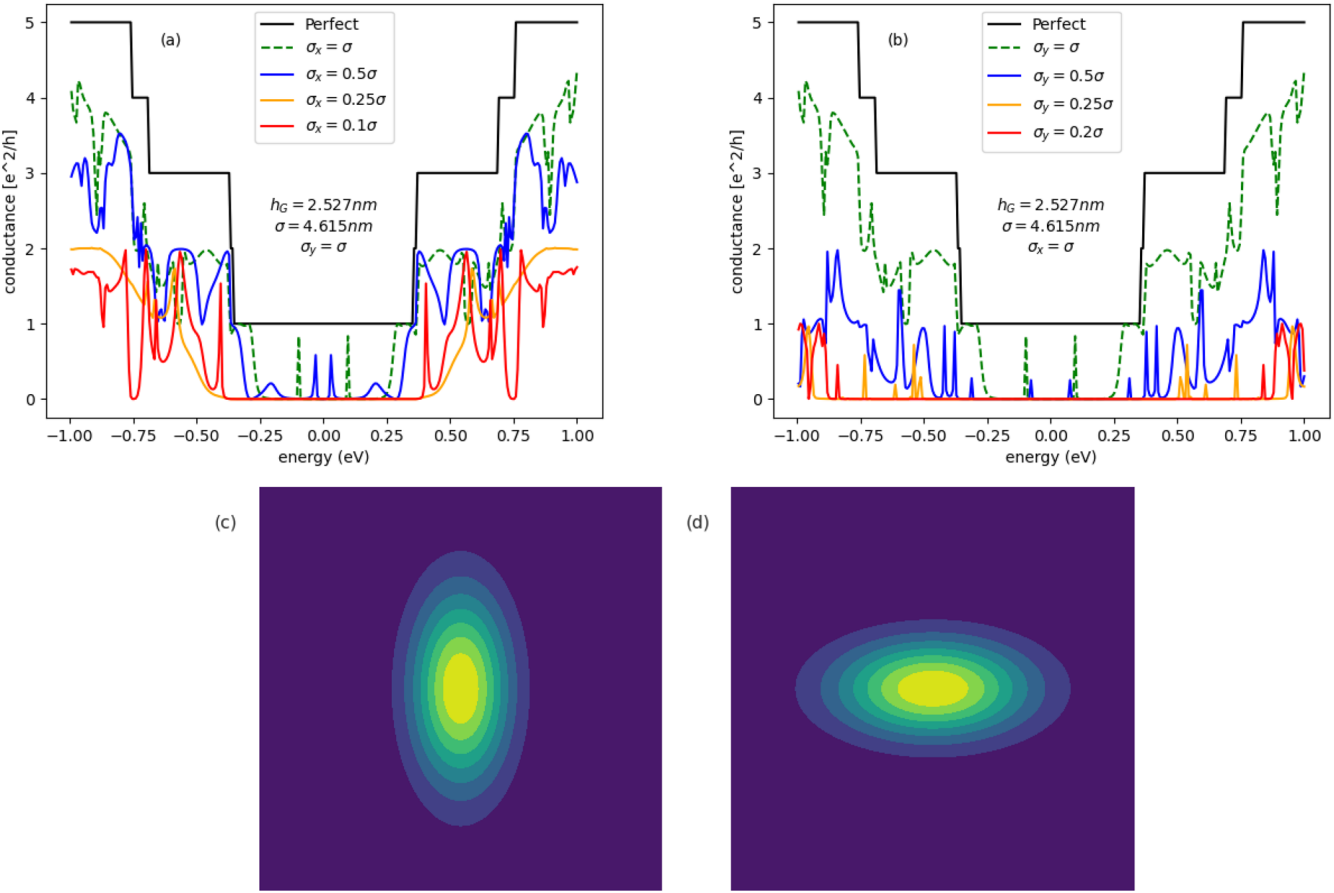
Conductance spectra of semimetalic armchair GNR with the central scattering region in form of deformed GQD ($M=41, N=200$) for several configurations of elliptic Gaussian deformation. Parameters of centrosymmetric Gaussian deformation ($h_G=2.527 \, \text {nm}, \sigma =4.615 \, \text {nm}$) are fixed on values considered in the paper^19^. Solid black lines correspond to perfect armchair GNR. Conductance spectra given in part (**a,b**) correspond to the kind of deformation sketched in part (**c,d**), respectively.

In Fig. 6 are given conductance spectra of semimetalic armchair GNR ($M=41$) with centrosymmetric Gaussian bump in the central region ($M=41, N=200$). For centrosymmetric case, the Gaussian bump parameters are fixed on values $h_G=2.527\,\text {nm}, \sigma =4.615\,\text {nm}$ corresponding to the strain intensity equal to $15\%$, considered in the paper^19^. Figure 6a present the evolution of the conductance with increasing size of the bump in the *x*-direction. The bump size in the *y*-direction is fixed on the value corresponding to the centrosymmetric case. We can see that due to elliptical deformation the energy gap increases with decreasing $\sigma _x$ and the peaks of the conductance in the gap region, appearing when the deformation is centrosymmetric, disappear. In Fig. 6b the dependence of the conductance on increasing size of the Gaussian bump, along the *y*-direction, is given. In this case, a larger band-gap is created, however the conductance is strongly suppressed outside the gap. Conductance spectra given in Fig. 6a,b correspond to the kind of deformation sketched in Fig. 6c,d, respectively.

**Figure 7:**
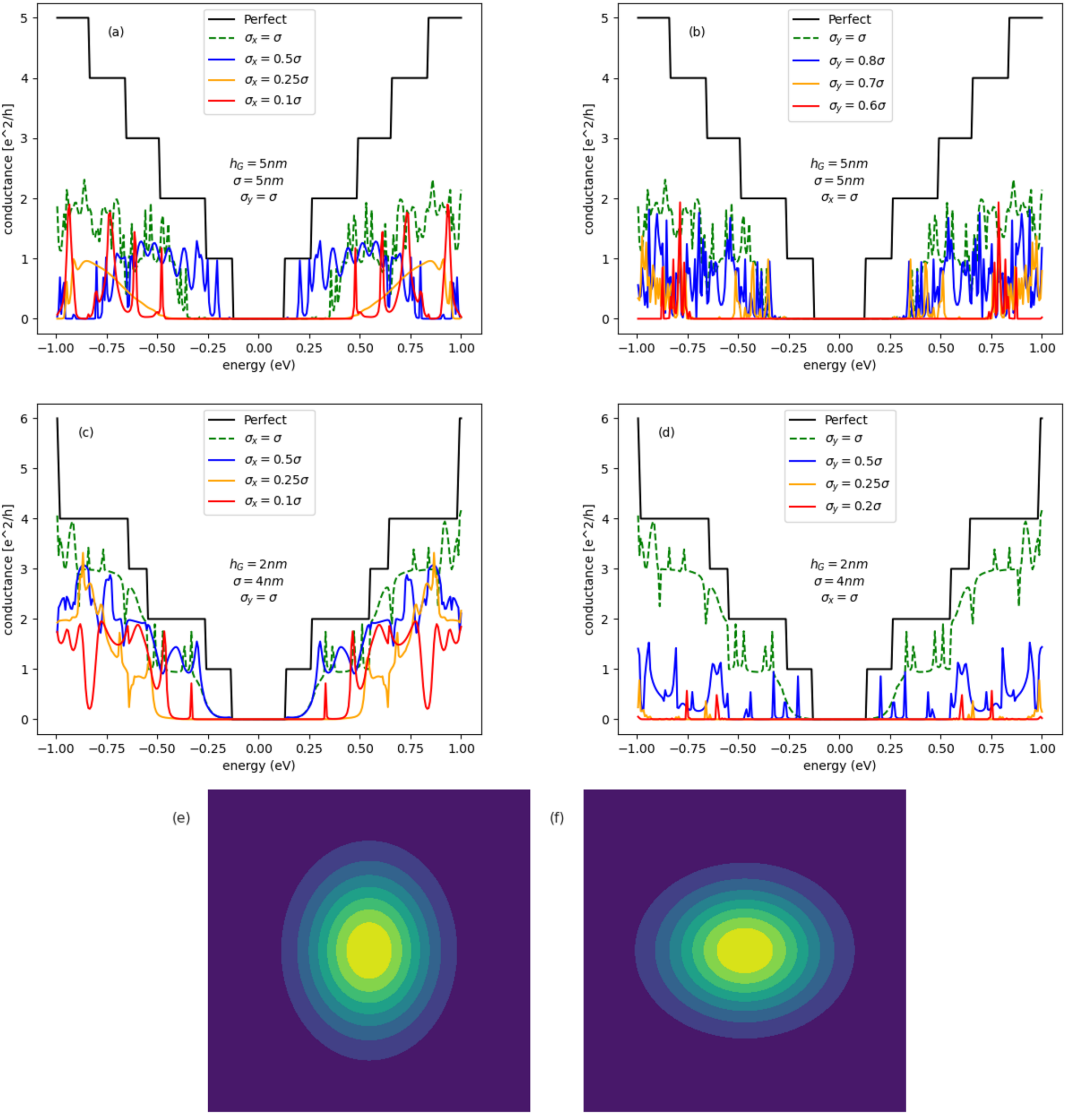
Conductance spectra of semiconducting armchair GNR with the central scattering region in form of deformed GQD: (**a,b**) ($M=39, N=300$); (**c,d**): ($M=37, N=300$), for several configurations of elliptic Gaussian deformation. Conductance spectra given in parts (**a,c**) (left panel) and (**b,d**) (right panel) correspond to the kind of deformation sketched in part (**e,f**), respectively.

In Fig. 7a,b are given conductance spectra of semiconducting GNR ($M=39$) with elliptically deformed GQD ($M=39, N=300$) in the center. The deformation parameter $\sigma _y$($\sigma _x$) is fixed and the conductance is given as a function of the parameter $\sigma _x$($\sigma _y$). We can see that both kinds of the deformation induce a wide transport gap, but simultaneously the conductance becomes weak outside the gap, in contrary to the system with centrosymmetric deformation. In Fig. 7c,d similar dependence are given for deformed semiconducting GNR ($M=37$). In this case a large band-gap is created but also, in opposite to the centrosymmetric case, the conductance is suppressed at higher energies. Conductance spectra given in Fig. 7a,c and Fig. 7b,d correspond to the kind of deformation sketched in Fig. 7e,f, respectively.

**Figure 8:**
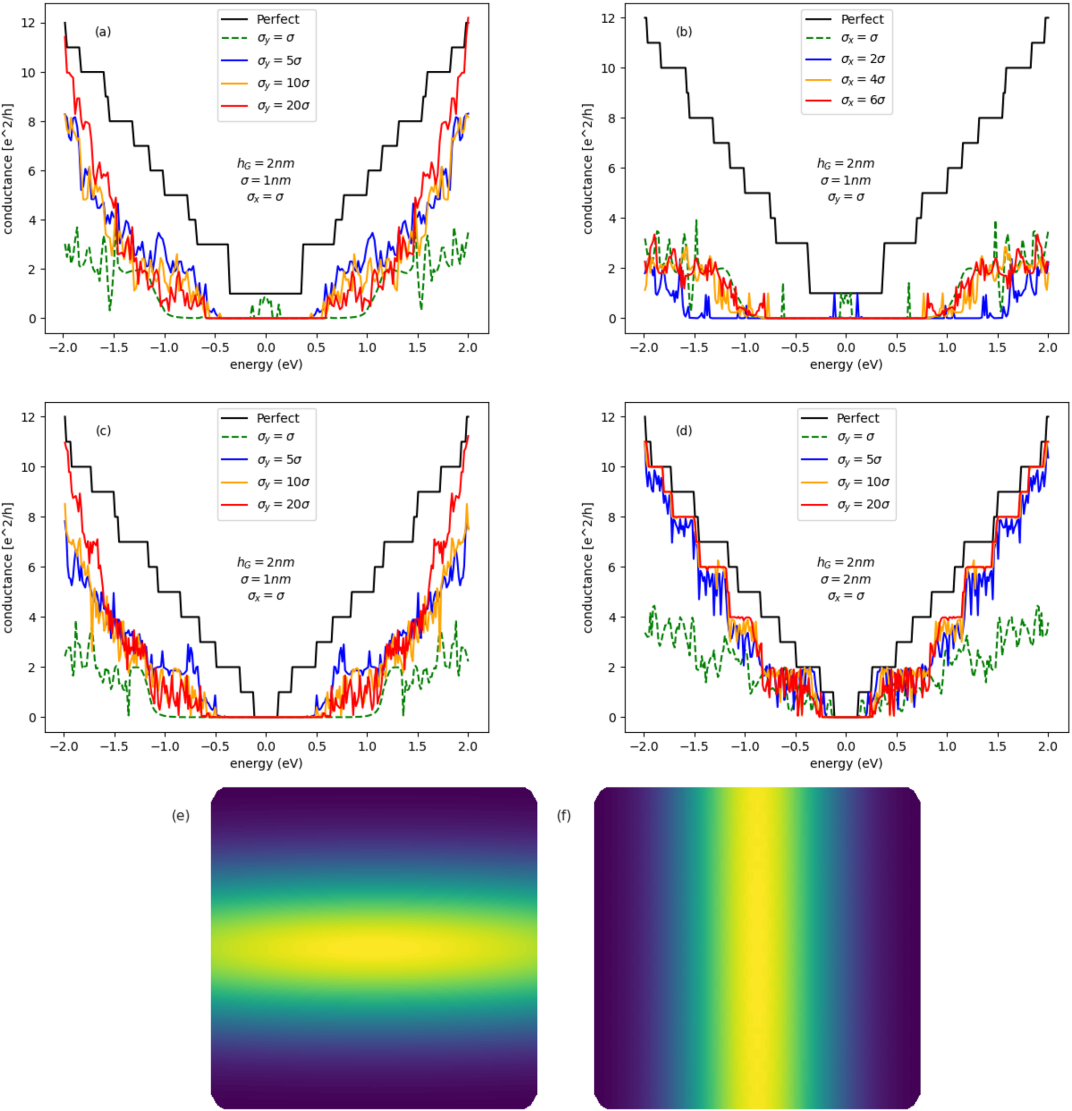
Conductance spectra of armchair GNR with the central scattering region in form of elliptically deformed GQD: (**a,b**) ($M=41, N=300$); (**c,d**) ($M=39, N=800$), for several configurations of Gaussian deformation. Conductance spectra given in part (**b**) correspond to the kind of deformation sketched in part (**e**). Conductance spectra given in parts (**a,c,d**) correspond to the kind of deformation sketched in part (**f**).

In Fig. 8a are given conductance spectra of semimetalic GNR ($M=41$) system. The Gaussian deformation of the central GQD is largely extended in one direction. The deformation generates the hopping energy profile given in Fig. 8f. We can see that in the case of the Gaussian fold oriented in the transport direction (large $\sigma _y/\sigma _x$ quotient) the transport gap is wider and the conductance becomes higher outside the gap, comparing to the centrosymmetric case. The conductance spectra given in Fig. 8b correspond to configuration given in Fig. 8e. This kind of the deformation leads to the large transport gap, however the conductance becomes very poor outside the gap. The conductance spectra of semiconducting systems ($M=39$) with the Gaussian deformation generating hopping profile given in Fig. 8f are presented in Fig. 8c,d. We can see in Fig. 8c that the case of Gaussian bump ($h_G/\sigma =2$) with low modulation in the transport direction $\sigma _y=20\sigma$ gives wide energy gap and good conductance for higher energies, comparable to the ballistic conductance of non-deformed GNR. On the other hand, as we can see in Fig. 8d, the system with lower deformation ($h_G/\sigma =1$) generates smaller band-gap, however the conductance becomes higher outside the gap. Both these configurations are optimal for practical electronic purposes, particulary from the point of view of sharp switching between *on* and *off* states in the field-effect nano-transistor, with GQDs built in.

## Summary

In this paper I studied static electronic properties and equilibrium transport properties of rectangular graphene quantum dots with non-centrosymmetric Gaussian deformation. Quantum transport has been studied within NEGF formalism for system with deformed GQD as a central scattering region connected to semi-infinite GNRs as leads, having perfect geometry. The connection between symmetry of spatial LDOS structure and the symmetry of local strain field has been observed, for a wide energy range. By investigating several types of deformation, I estimated optimal configuration of the armchair GNR system with elliptic Gaussian deformation, suitable for potential electronic applications. The optimal configuration corresponds to the extended elliptic deformation having a geometry of a *gutter* with decreasing depth, oriented along the transport direction. Contrary to the centrosymmetric bumps or strictly Gaussian-fold deformations considered in many papers, this kind of the deformation provides a large transport gap and high transmission outside the gap, that is comparable with the transmission of perfect GNRs. These two features are fundamental from the point of view of practical nanoscale electronics applications. In the case of field-effect transistors in particular, the achievement of a high on/off ratio is directly dependent on these characteristics. One should be also added that discussed in this paper cases are representative examples of whole family of armchair GNR systems having the form GNR-GQD-GNR and obtained results are universal in the sense that they do not depend on particular integer number M, defining one of the three family members.

## Supplementary Information

Supplementary Information.

## Data Availability

The datasets used and/or analyzed during the current study are available from the corresponding author on reasonable request.
