# Supplementary material for: Electron transport properties of graphene quantum dots with non-centro-symmetric Gaussian deformation
Source: Sci Rep. 2022 Jun 14;12:9908. doi: 10.1038/s41598-022-14251-5 (PMC9198241; doi:10.1038/s41598-022-14251-5)
Supplement: Supplementary file 1 — Supplementary Information. [file 41598_2022_14251_MOESM1_ESM.pdf]

Appendix: The LSF

According to Eq. (2) strain effects are included via modification of the off-site TB parameters. We can introduce the concept of local strain field (LSF) by averaging the hopping parameter along the contour given in Figure A1, centred on the position (x, y) treated as continuous variable. We note that the contour is the projection on the xy plane of closed 3D curve lying on the surface of deformed GQD. The hopping energy between two sides i and j is given by Eq. (2),

$$t_{ij} = t_0 e^{-\beta(d_{ij}/a_0 - 1)} \tag{A.1}$$

where $d_{ij} = \sqrt{(x_i - x_j)^2 + (y_i - y_j)^2 + (z_i - z_j)^2}$. The height of atoms is given by Eq. (3) with $(x_0, y_0) = (0, 0)$,

$$z_k(x, y) \equiv z(x_k(x, y), y_k(x, y)) = h_G e^{-[(x_k(x,y))^2/2\sigma_x^2 + (y_k(x,y))^2/2\sigma_y^2]}, \tag{A.2}$$

where the index k corresponds to the k-th vortex of the hexagon $(k = 1, ..., 6)$. Since $z_k = z_k(x, y)$ is the function of coordinates of the center of the hexagon, the distances d_{ij} and hoppings t_{ij} also are and we can define the locally averaged hopping energy field,

$$\mathcal{T}(x, y) = \frac{1}{6}[t_{16}(x, y) + t_{21}(x, y) + t_{32}(x, y) + t_{43}(x, y) + t_{54}(x, y) + t_{65}(x, y)], \tag{A.3}$$

where hoppings t_{ij} are taken between adjacent vortices along the closed hexagonal contour, centred around the position (x, y) treated as continuous variable.

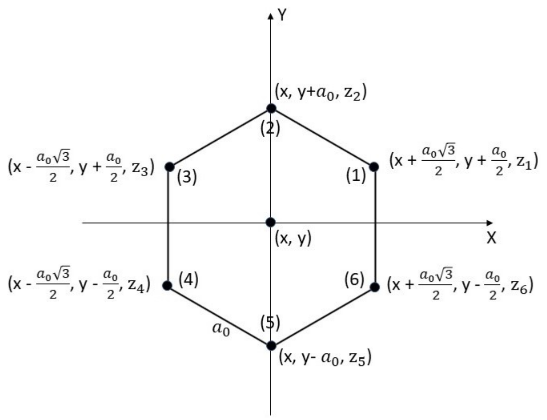

Fig.A 1: The projection on xy plane of 3D hexagonal contour around the continuous position (x, y) on the surface of GQD with out-of-plane Gaussian deformation.
